# Ventricular Flow Field Visualization During Mechanical Circulatory Support in the Assisted Isolated Beating Heart

**DOI:** 10.1007/s10439-019-02406-x

**Published:** 2019-11-18

**Authors:** P. Aigner, M. Schweiger, K. Fraser, Y. Choi, F. Lemme, N. Cesarovic, U. Kertzscher, H. Schima, M. Hübler, M. Granegger

**Affiliations:** 1 Center for Medical Physics and Biomedical Engineering, Medical University of Vienna, Waehringer Guertel 18-20, AKH-4L, 1090 Vienna, Austria; 2 Ludwig Boltzmann Institute for Cardiovascular Research, Vienna, Austria; 3 Department of Mechanical Engineering, University of Bath, Bath, UK; 4 Pediatric Cardiovascular Surgery, Department of Surgery, Pediatric Heart Center, University Children’s Hospital Zurich, Zurich, Switzerland; 5 Children’s Research Center, University Children’s Hospital Zurich, Zurich, Switzerland; 6 Division of Surgical Research, Department of Surgery, University Hospital Zurich, University of Zurich, Zurich, Switzerland; 7 Biofluid Mechanics Laboratory, Institute for Imaging Science and Computational Modelling in Cardiovascular Medicine, Charité-Universitätsmedizin Berlin, Berlin, Germany

**Keywords:** Echocardiographic particle image velocimetry, Mechanical circulatory support, Left ventricular assist device, Ultrasound velocimetry

## Abstract

Investigations of ventricular flow patterns during mechanical circulatory support are limited to *in vitro* flow models or *in silico* simulations, which cannot fully replicate the complex anatomy and contraction of the heart. Therefore, the feasibility of using echocardiographic particle image velocimetry (Echo-PIV) was evaluated in an isolated working heart setup. Porcine hearts were connected to an isolated, working heart setup and a left ventricular assist device (LVAD) was implanted. During different levels of LVAD support (unsupported, partial support, full support), microbubbles were injected and echocardiographic images were acquired. Iterative PIV algorithms were applied to calculate flow fields. The isolated heart setup allowed different hemodynamic situations. In the unsupported heart, diastolic intra-ventricular blood flow was redirected at the heart’s apex towards the left ventricular outflow tract (LVOT). With increasing pump speed, large vortex formation was suppressed, and blood flow from the mitral valve directly entered the pump cannula. The maximum velocities in the LVOT were significantly reduced with increasing support. For the first time, cardiac blood flow patterns during LVAD support were visualized and quantified in an *ex vivo* model using Echo-PIV. The results reveal potential regions of stagnation in the LVOT and, in future the methods might be also used in clinical routine to evaluate intraventricular flow fields during LVAD support.

## Introduction

Over the past years mechanical circulatory support (MCS) therapy has progressively improved. Two-year survival rates of patients with rotary blood pumps (RBP) used as left ventricular assist devices (LVAD) have increased to almost 80%.^28^ However, adverse events such as thromboembolism and major bleeding complications hinder the further success of the therapy.^28^ These adverse events have been attributed to the altered and non-physiologic blood flow patterns within the pump and/or the altered flow field within the assisted ventricle.^3^,^4^,^36^,^45^,^46^

In the healthy heart, complex three-dimensional diastolic vortical flow grants washout of the entire ventricle.^10^ Once blood enters the ventricular cavity, it traverses in a large clockwise vortex from the cardiac base to the apex, which then is directed towards the left ventricular outflow tract (LVOT).^51^ Energy dissipation is reduced by this vortical flow pattern by avoiding rapid accelerations and gradients; thus, improving contractile efficiency.^1^,^2^,^10^,^42^ Since alterations in these vortical flow patterns are associated with different pathological states, they may be even used as a predictor for impending cardiovascular diseases.^19^,^22^,^23^,^39^

Despite its unquestionable clinical benefit, LVADs disturb the physiologic interplay of the cardiac structures and blood flow in two ways: First, the inflow cannula protrudes into the left ventricle, creating a geometric obstacle to the native diastolic flow patterns. In the apical region, wedge thrombus formation around the cannula was attributed to the combined effect of the altered flow field and geometric obstruction.^20^,^25^,^49^ Second, rotary LVADs draw blood from the apex throughout the entire cardiac cycle, disrupting native vortex formation.

The degree of pump support is determined by the pump speed and the current cardiovascular condition of the patient. Either the entire cardiac output is provided through the LVAD while the aortic valve remains closed—full support—or the LVAD supplements the cardiac output through the aortic valve by pumping in parallel with the left ventricle—partial support. The flow field and pulsatility in the LVOT is especially altered during full support mode.^45^ This unphysiologic flow field in the LVOT, which is suspected to contribute to valve stenosis and insufficiencies, may also be responsible for thrombus formation.^27^ These thrombi may detach and are a considerable contributor towards the adverse events associated with LVAD therapy.^20^

To investigate the ventricular flow patterns in LVAD patients, imaging modalities that enable visualization of these flow fields *in vivo* are required. The typical method of using phase contrast 4D MRI for flow field visualization^9^,^34^ cannot be applied in LVAD patients due to the ferromagnetic parts. Echocardiography is clinically used to monitor cardiac dimensions and functionality but is limited to unidirectional flow velocity measurements.^15^,^50^*In vitro* models are able to capture typical flow field structures; however, they involve simplification of complex ventricular structures such as the mitral valve apparatus and ventricular motion.^36^,^45^ Numerical simulations are also limited as they inherently rely on geometric and hemodynamic simplifications and cannot capture the whole complexity and interaction of the blood flow with the heart.^7^,^8^,^31^,^51^

Therefore, the aim of this study was to investigate the left ventricular flow field of an isolated beating heart with an LVAD implanted using echocardiographic particle imaging velocimetry (Echo-PIV).

## Materials and Methods

### The Isolated Heart

Hearts of 4 landrace pigs (80–106 kg) were explanted according to the protocol described previously.^22^ Briefly, a median sternotomy was performed in the anesthetized pigs and the aorta was cannulated with a cardioplegic needle. After exsanguinating around 2 L of blood *via* the iliac artery, the ascending aorta was clamped distally to the cardioplegic needle and 1 L of Custodiol (Dr. Franz Köhler Chemie GmbH, Bensheim, Germany) was slowly applied. Simultaneously, the heart was cooled with a slurry ice solution. The heart was then excised and prepared for the connection to the isolated heart apparatus. All vessels of the right atrium were ligated using 4-0 Prolene sutures. All vessels of the left atrium were also closed except one pulmonary vein that was connected to the preload reservoir. The HVAD (Medtronic plc, Dublin, Ireland) sewing ring was attached to the ventricular apex to facilitate coring and pump implantation with orientation of the LVAD inflow cannula towards the mitral valve.

**Figure 1:**
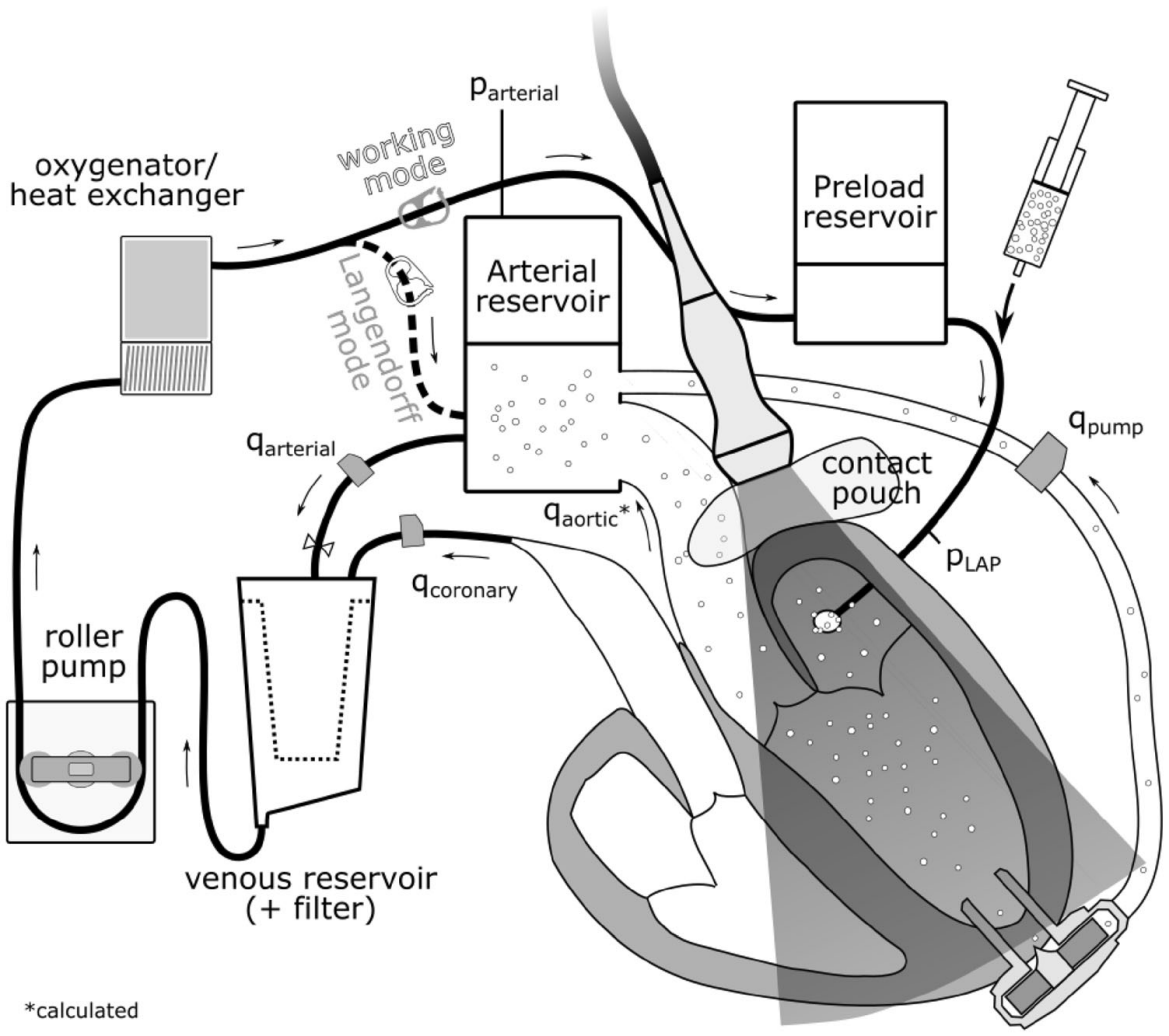
Schematic of the isolated heart apparatus in working mode. The Echo probe could be placed anywhere on the cardiac surface and allowed unlimited views for echocardiographic particle image velocimetry.

The isolated heart apparatus featured three connections to the heart: First, the aorta was connected to an air-trapped reservoir mimicking the arterial Windkessel^53^ system; Second, the pulmonary artery was connected to an open venous reservoir to eject the coronary flow against a low pressure; Third, the left atrium was cannulated and connected to a pressure-controlled air-trapped reservoir which constituted the preload adjustment system (Fig. 1).

For cardiac resuscitation, Langendorff perfusion was used as the pressure in the arterial reservoir was gradually increased until 70 mmHg was reached.^22^ Thereby, the coronary arteries were perfused with oxygenated blood and the heart was rewarmed to a normothermic temperature of 37 °C. If necessary, the hearts were defibrillated and/or paced to achieve a stable sinus rhythm. Once the heart stabilized, an HVAD was attached and the setup was switched to the working mode, in which the heart pumps blood from the preload reservoir towards the arterial one. By adjusting the preload, afterload, and pump speed, different levels of LVAD support (unsupported, full, and partial support) were achieved. Characteristic pressure (APT300, Harvard Apparatus, Hollisten, MA, USA, see Fig. 1) and flow signals (SONOFLOW CO.55/120, Sonotec, Halle, Germany, see Fig. 1) were recorded with a signal processing and control board (MicroLabBox, dSPACE GmbH, Paderborn, Germany).

### Echocardiographic Image Acquisition

For flow visualization purposes, B-mode echocardiography images of the left ventricle in long-axis view were acquired (Philips iE33, X5-1 xMatrix probe, Philips Electronics N.V., Bothell, WA, US). Measurements were performed by placing the echo-probe either on the sagittal portion of the left atrium or on the lateral surface of the right heart. By using the atrial approach, the required width of the echo-images was generally narrower, and therefore, the maximum frame rate in this view was higher. To enhance the visualized sector with a minimum number of sweeping lines, a thin-walled fluid-filled silicone pouch was used to increase the distance between the echoprobe and the epicardial wall. Additionally, the silicone pouch improved contact with the atrial wall as it partially compensated for the motion of the atrial contraction. With the resultant small sector angle, it was possible to capture the whole left ventricle with a high frame rate of 99 to 137 Hz. High frame rates are vital for capturing the high velocity magnitudes present since the displacement of the microbubbles must be small enough that they remain within the same PIV interrogation region of an image pair.

**Figure 2:**
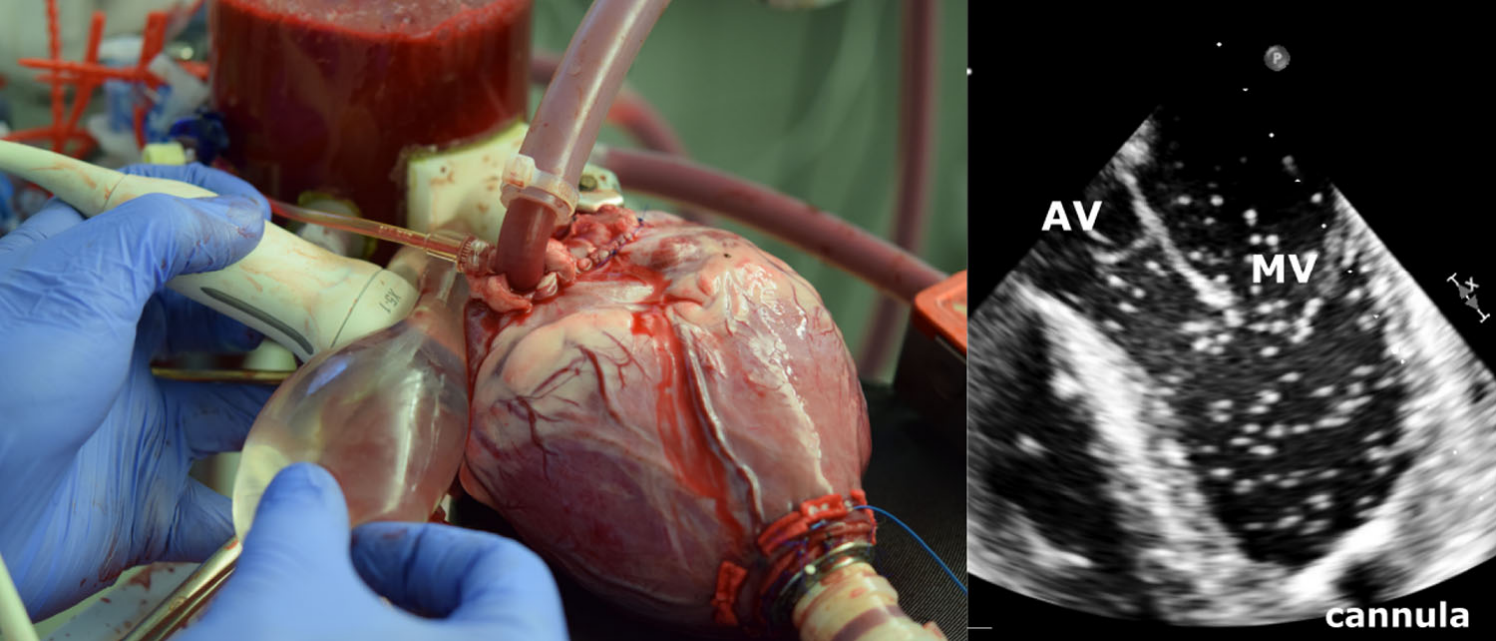
Left: use of the fluid-filled silicone pouch for improved contact with the cardiac surface and compensation of cardiac motion. Right: exemplary echocardiography image of the left ventricle during application of the microbubbles.

A microbubble solution was injected into the preload reservoir, from where it was immediately transported into the left ventricle and visualized in the B-mode images (Fig. 2). The microbubbles were created by forcefully agitating saline and air (5–10 vol% air) between two syringes connected with a three-way stopcock for at least five times 5,16,26,35 until the solution became partly opaque and white. For each heart, B-mode sequences in DICOM format were recorded for an unsupported, partial, and full support condition.

### Echo PIV

The B-mode images were analyzed using a previously validated iterative PIV algorithm.^18^,^41^ The algorithm consisted of two stages: First, a region of interest was selected in the LV outflow tract and a simple 2D cross-correlation was applied on this region for all image pairs from 3 cardiac cycles. This cross-correlation was used to give a rough estimate for the velocity magnitude in that region, which also provided a periodic signal with the same periodicity as the cardiac cycle. This signal was used to split the echo data into single cardiac cycles, align those cycles, and set up data bins for each 1/50th phase of a cardiac cycle. Based on preliminary evaluations, the use of three consecutive cardiac cycles was found to be sufficient. Further, even in case of arrhythmia it was possible to record three consecutive beats with the same duration in all experiments. The second stage was an iterative PIV algorithm which used all the image pairs within a single bin. Coarse regions of interest (48 by 48 pixels with 50% overlap) were first used to calculate cross-correlations which were then averaged. These averaged cross-correlations gave a rough vector field, that was used to apply a displacement map to the locations of the finer regions of interest (24 by 24 pixels with 50% overlap) within the second image of each image pair such that the particles remained within the regions of interest. The final vector field consisted of 53 by 42 pixel maps with each region being approximately 3 by 3 mm depending on the field of view for the whole cardiac cycle.

Calibration of dimensions was based on the scaling factor (image resolution 800 by 600 pixels, spatial resolution 0.15–0.26 mm/px) in the DICOM data header. A sweep correction algorithm^18^ was additionally applied to compensate for errors resulting from the echo-image creation by sweeping the echo beam through the imaging plane.

The flow patterns for each different support level and experiment were visualized. The mean velocity flow field over one cardiac cycle (steady streaming analysis) was computed to identify general flow features. Further, velocities in the LVOT were quantified for each of the experiments. The normalized flow pulsatility in the LVOT was computed by the difference between the maximum and minimum velocity (*v*_max_, *v*_min_) normalized over the mean velocity (*v*_mean_, Eq. 1) to compensate for cardiac output variability across experiments.

1 $${\text{Normalized}}\;{\text{Pulsatility}} = \frac{{v_{\hbox{max} } - v_{\hbox{min} } }}{{v_{\text{mean}} }}$$

### Statistical Comparison

The statistical analysis was performed using SPSS for Windows Release 23.0.0 (SPSS Inc. Chicago, IL, USA). Metric variables are reported as median and range. One-way analysis of variance followed by a Scheffé post hoc comparison were employed to determine statistical significance levels among support groups. A *p*-value < 0.05 was considered statistically significant.

## Results

### Hemodynamics

Four hearts were successfully explanted (animal weight: 87.3 ± 5.2 kg, heart weight: 354.5 ± 24.5 g) and connected to the isolated heart apparatus. All of them were resuscitated without complications within 55 ± 12 min after cardiac activity ceased. Different hemodynamic situations within the isolated heart setup were adjusted and recorded for the three support conditions (unsupported, partial support, full support). The range of the hemodynamics is summarized in Table 1.

**Table 1:** Hemodynamic range found in the Echo-PIV experiments reported as median (range).

Parameter	Unsupported (*n* = 3)	Partial support (*n* = 7)	Full support (*n* = 5)	*p* (ANOVA)
Mean arterial pressure (mmHg)	55.8 (48.1–60.0)	70.5* (63.0–83.1)	70.4* (69.0–72.5)	**0.002**
Left atrial pressure (mmHg)	29.9 (23.1–33.9)	24.3 (20.6–31.0)	19.3*^+^ (11.1–21.0)	**0.011**
Pump flow (L/min)	–	3.90 (2.15–4.94)	4.21 (3.70–4.94)	0.23^#^
Coronary flow (L/min)	0.70 (0.69–0.80)	0.94 (0.70–1.10)	1.00* (0.95–1.29)	**0.009**
Arterial flow (L/min)	2.36 (0.83–2.37)	3.13* (2.56–4.27)	3.18 (2.59–3.34)	**0.025**
Aortic valve flow (L/min)	3.05 (1.50–3.14)	0.59* (0.21–1.35)	− 0.08* (–0.40–0.19)	**< 0.001**
Pump speed (rpm)	–	2400 (2100–2500)	2500 (2400–2800)	**0.03**^**#**^
Heart rate (bpm)	83 (39–93)	82 (42–93)	74 (58–83)	0.911

Bold values indicate statistical significance (*p* < 0.05)

*p < 0.05 compared to unsupported condition (Scheffé test)

^+^*p* < 0.05 compared to partial support condition (Scheffé test)

^#^Two-group comparison using 2-sided t-test

### Flow Fields

#### Time Averaged Flow Analysis

**Figure 3:**
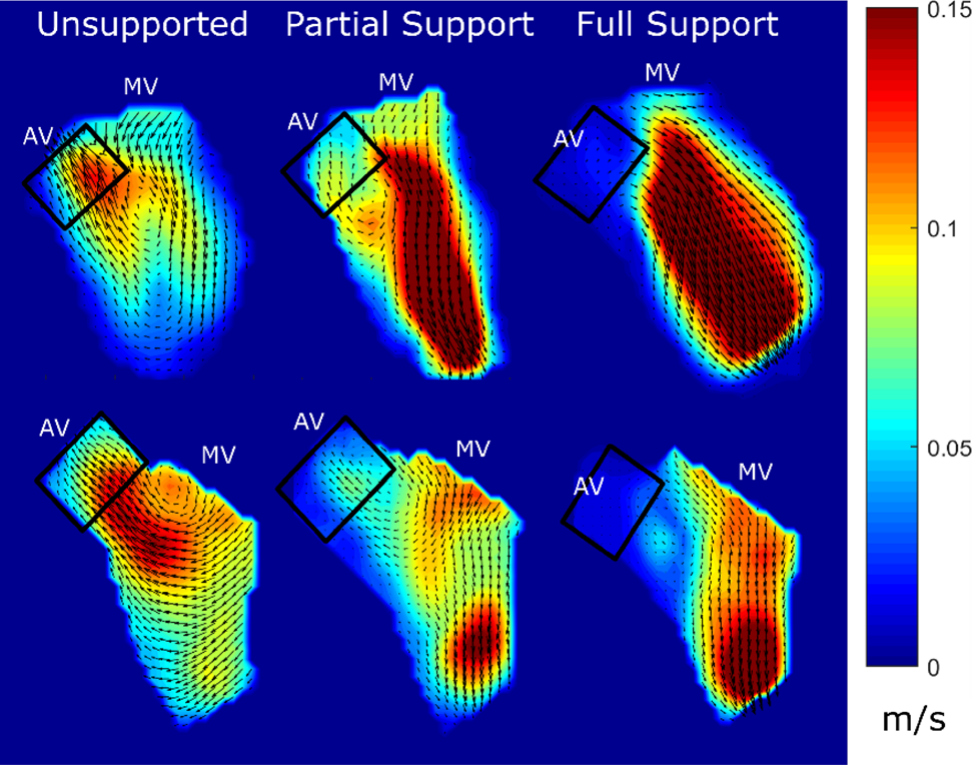
Steady streaming analysis (time averaged mean velocities) of two hearts at different support levels (left) unsupported, (middle) partial support, (right) full support; *AV* aortic valve; *MV* mitral valve.

To visualize the key features of the ventricular flow fields in the three investigated conditions, a steady streaming analysis (time averaged mean velocities over the whole cardiac cycle) was performed (Fig. 3). Velocities in the LVOT were reduced with increasing LVAD support and a flow channel guiding blood from the mitral valve directly towards the LVAD inflow cannula was created. Comparing the unsupported and full support conditions, there was an observed shift of the peak velocities in the LVOT towards the region close to the ventricular free wall and the apex.

**Figure 4:**
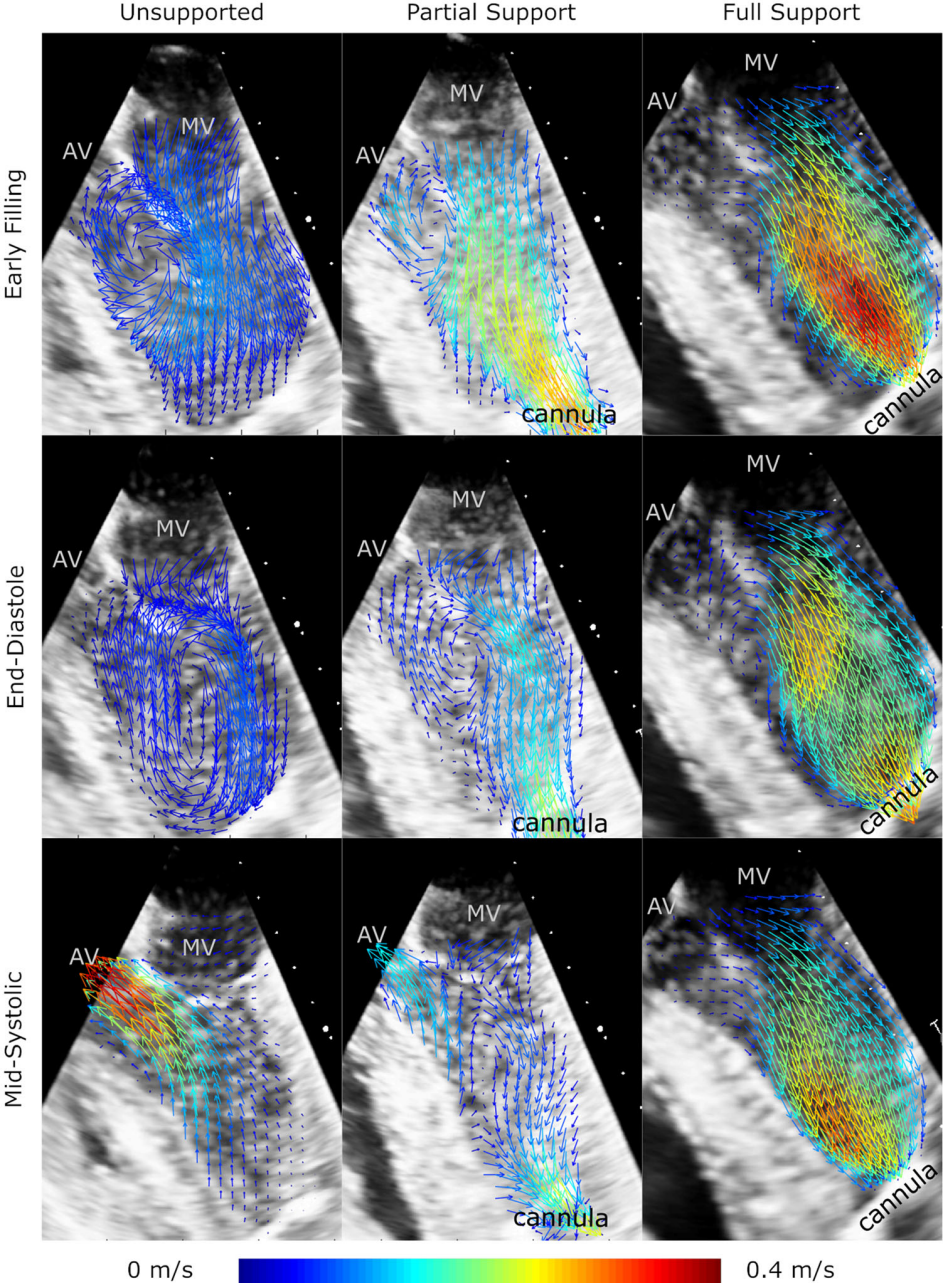
Flow fields within the left ventricle for three cardiac phases from the atrial view with the LVAD cannula at the bottom of the image (vectors indicating the flow direction, vector velocities color coded).

In a next step, to quantitatively assess the flow in the LVOT, a time resolved analysis was performed in the areas bound by black rectangles in Fig. 4.

#### Instantaneous Flow Analysis

**Figure 5:**
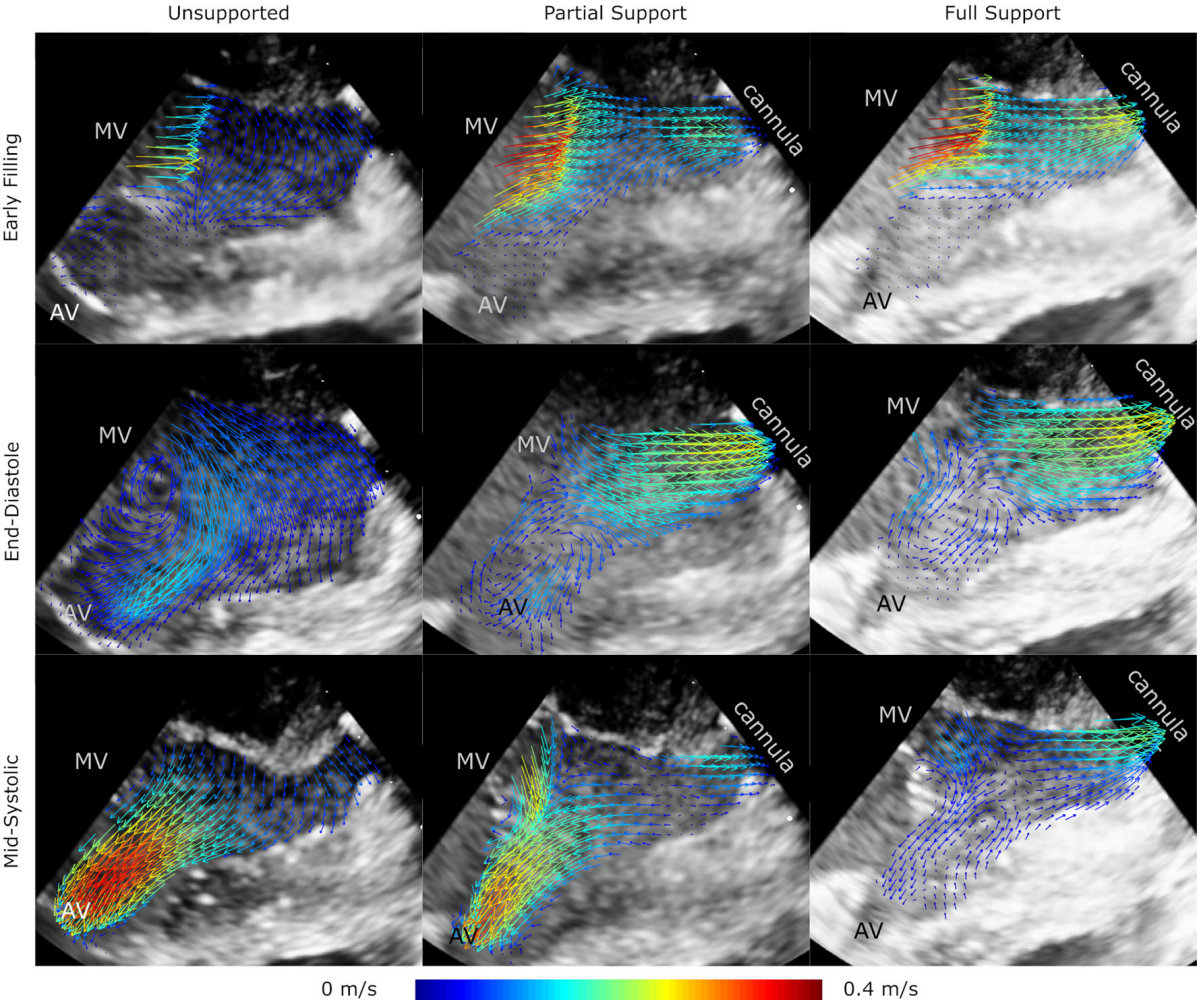
Flow fields within the left ventricle for three cardiac phases from the lateral view with the LVAD cannula at the right side of the image (vectors indicating the flow direction, vector velocities color coded).

In unsupported hearts, the following ventricular flow patterns were observed. In the early diastolic filling phase, a clockwise vortical structure was created by the mitral inflow starting at the LVOT (left column in Figs. 4 and 5). Until the end of the diastole, this vortical structure enlarged until it encompassed the entirety of the ventricular cross section. The diastolic mitral inflow was hence redirected towards the LVOT through this enlarged vortical flow structure that extended down to the apex in a clockwise direction. During systolic ejection through the aortic valve, peak velocities reached 0.50 (0.43–0.54) m/s and the vortical flow structure vanished. The mean velocity over the cardiac cycle in the LVOT was 0.08 (0.07–0.09) m/s (Table 2).

**Table 2:** Statistical evaluation of velocity data in the LVOT gathered in atrial and lateral view; data presented as median (range)

Parameter	Unsupported (*n* = 3)	Partial support (*n* = 7)	Full support (*n* = 5)	*p* (ANOVA)
*v*_min_ (m/s)	0.00015 (0.0000–0.002)	0.00043 (0.0001–0.0014)	0.00024 (0.0001–0.0006)	0.208
*v*_max_ (m/s)	0.50 (0.43–0.54)	0.27* (0.07–0.47)	0.14* (0.06–0.17)	**0.001**
*v*_mean_ (m/s)	0.08 (0.07–0.09)	0.06 (0.01–0.07)	0.03* (0.02–0.06)	**0.009**
*v*_max_/*v*_min_	3637 (1763–34189)	567 (155–1425)	735* (188–1216)	0.068
Normalized pulsatility (*v*_max_ − *v*_min_)/*v*_mean_	5.3 (5.1–7.8)	5.1 (2.8–8.2)	3.8*^+^ (3.1–5.4)	0.198

Bold values indicate statistical significance (*p* < 0.05)

*p < 0.05 compared to unsupported condition (Scheffé test)

^+^*p* < 0.05 compared to partial support condition (Scheffé test)

Marked differences were observed between the unsupported and partially supported ventricles (middle column in Figs. 4 and 5). The diastolic vortex formation, similar to the unsupported condition, formed in the LVOT during the early filling phase; However, the vortex enlarged to a lesser extent than in the unsupported heart due to the LVAD acting as a flow sink as it continuously drained blood from the apex. This reduced the size of the vortex such that it did not encompass the entire ventricular cross section. The peak velocities in the LVOT were significantly reduced compared to the unsupported situation (mean 0.06 (0.01–0.07) m/s and peak 0.27 (0.07–0.47) m/s) (Table 2).

During full support (right column in Figs. 4 and 5), the aortic valve was continuously closed and the physiologic vortical formation and flow structures were absent. Although clockwise vortical structures were formed towards the end of diastole in the LVOT, they did not encompass the entire ventricular cross section. The velocities in the LVOT were considerably lower (mean velocity 0.03 (0.02–0.06) m/s and peak velocity 0.14 (0.06–0.17) m/s) compared to the partial support condition. Due to the closed AV during the entire heart cycle, the pulsatility during the heart cycle was significantly reduced compared to the partial support condition. A detailed comparison of velocities and pulsatility in the LVOT is provided in Table 2.

## Discussion

The impact of an implantable RBP on intra-ventricular blood flow is suspected to substantially contribute to the high rate of thromboembolic adverse events observed in LVAD patients.^37^ However, this interaction cannot be investigated *in vivo* with standard clinical imaging methods like echocardiography or MR-scans. In *in silico* and *in vitro* models, the LV and its interaction with the mitral valve apparatus cannot be evaluated in its full complexity (e.g. limited to bioprosthetic valves,^45^ non-contractile,^7^ or non-functional mitral valve^31^). In this study, the isolated beating heart setup and Echo-PIV methods coalesced into a tool to investigate the flow patterns in the ventricle without the need of extrapolations and/or simplifications of any cardiac structure intrinsic to other models.

Ventricular flow fields in healthy and dysfunctional ventricles without LVAD support have previously been evaluated by using Echo-PIV.^1^,^10^ Reports on the effect of LVAD support on cardiac flow patterns in a realistic setting are lacking. In this study, the isolated heart provided a superior setting compared to usual animal experiments in terms of highly tunable hemodynamic conditions and accessibility for echocardiographic imaging from any direction. The acquisition of echocardiographic recordings and the application of the previously validated Echo-PIV algorithms with frame rates of ≥ 99 Hz was facile and performed without the use of specific echo contrast agents, as were employed previously.^16^,^26^ Buoyant microbubbles with a diameter of ~ 25 *µ*m were used,^26^ which were small enough to follow the flow. Based on Stokes’ drag law,^44^ the gravitationally induced velocities (0.0001 m/s) were negligible compared to the measured velocities.

To set different hemodynamic situations the isolated heart setup provided several parameters such as preload, afterload, roller pump flow, LVAD speed, and medication to adjust contractility. To realize the transition from partial support to full support the LVAD speed was increased. Thereby a shift of the flows was seen with more LVAD flow and less aortic valve flow. At the same time the preload (left atrial pressure) slightly dropped caused by the higher pump speed as seen in Table 1. The achieved hemodynamic conditions were comparable to clinical values reported pre- and post-LVAD implant^24^ and recommended by MCS guidelines.^17^,^48^

In the unsupported heart, similar flow patterns and velocities were found as reported by previous studies.^1^,^2^,^10^ In the LVOT, the mitral inflow generated a clockwise rotational pattern, which was initially located by the anterior mitral valve leaflet. Towards end-diastole, that vortex enlarged until it covered the entire left ventricular area in clockwise direction. This mechanism supposedly stores kinetic energy during diastole to enhance cardiac efficiency in healthy hearts.^19^ A second vortex usually found under the posterior mitral leaflet in healthy hearts^6^ was absent and indicates that the unsupported hearts are not representative of healthy ventricular function, but instead depicts patterns similar to what is seen in dilated hearts.^38^,^40^ In *in vitro* studies, the formation of this vortex presented differently as the LVOT-mitral valve apparatus could never be modelled realistically but the reported velocities were in the same range.^30^,^45^,^54^ Consequently *in vitro* rotational patterns formed closer to the chamber center and the interaction with the LVOT can hardly be modelled *in vitro*.

In partial support, the cardiac output was split into the pump flow and the flow transferred *via* the regular pathway—the AV. Thereby, the formation of the clockwise structure was diminished as the flow from the mitral valve to the inflow cannula was partially channeled into the pump which limited the area of the clockwise vortical structure in the ventricular area. Although this led to different interventricular flow fields, washout of the LVOT seemed warranted. Pulsatility and velocities were reduced, but only low during the diastolic portion of the cardiac cycle. Velocities in the LVOT were easily measureable and the formation of the large vortical structure started behind the anterior mitral leaflet, inferior of the AV similar to the unsupported situation. This patterns could not be shown in experimental studies.^30^,^45^,^54^

In full support, flow pulsatilities were drastically reduced during the entire cardiac cycle. The mitral inflow was immediately directed toward the LVAD inflow cannula leading to unphysiologic flow along the long axis of the ventricular chamber. The velocities in the LVOT and the formation of a large vortical structure was noticeably diminished. The flow direction in the LVOT was even inconsistent with some cases showing a counter-clockwise rotation. In all experiments, the LVOT velocities in full support were below 0.06 m/s, which poses a risk for stagnation. Clinically, it is known that this area is prone to thrombus formation. The apparent low velocities further substantiate the connection between no aortic valve opening and prevalence of thromboembolic events,^8^,^13^,^36^,^37^,^46^ which was also demonstrated in other experimental and simulation studies.^33^,^43^,^45^,^52^

To prevent thrombus formation in these regions, LVAD research has focused on periodic or synchronous pump speed changes^21^,^29^,^32^,^52^,^55^ to promote aortic valve opening and minimize potential stagnation areas within the ventricle and the pump.^21^,^29^,^32^,^55^ However, the required amplitude, frequency, and clinical applicability of these speed changes to ensure proper washout in the LVOT remains unknown. The presented methodology presents a uniquely suitable tool for such investigations. The interaction of the cardiac valves with dynamic pump speed profiles and the effects on the flow field in the LVOT under realistic hemodynamic conditions can be evaluated not only towards thrombosis risk but also on valve function.

## Outlook

The presented method and findings of this study are a first step towards a standardized approach to assess flow fields within supported ventricles for research and clinical purposes. Currently, flow fields were calculated through post processing and were not available for real-time monitoring during the experiment. Nevertheless, the use of Echo-PIV is a promising technology that may be used in the future during LVAD implantation, where transesophageal echocardiography is clinical routine. Proper visualization of the left ventricle could be done with clinically approved contrast agents and real-time Echo-PIV flow pattern analysis. This could enable this technique to be used in the operating theatre to optimize pump speed and position.

This work focused on the feasibility of the method and there are still some challenges that need to be overcome in future. Further studies are required to define the same plane in each heart in order to quantify and statistically analyze the flow fields in more detail or even use 3D echocardiography with a better temporal resolution.

## Limitations

A limitation of porcine hearts are the anatomical differences between healthy porcine and human hearts, which needs to be considered in the interpretation of the results.^11^ Whereas this limitation may be acceptable for the investigation of the LV flow field, the flow structures around the inflow cannula in a dilated ventricle could not be assessed. Therefore, for studies focusing on improved cannula shape, position, and optimized length, the use of dilated ventricles is currently irreplaceable,^47^,^49^ which would require the induction of heart failure in long term animal experiments.^12^

With the limited frame rate of Echo-PIV, reliable results can be obtained for velocities below 0.7 m/s^18^,^41^ and high velocities might be underestimated. In this study, all velocities were below this limit because of relatively low flow rates. However, if the intraventricular flow field in e.g. an exercise condition was investigated, higher velocities would be expected requiring higher frame rates.

The Echo-PIV measurements allowed 2D flow pattern analysis in one plane. Although vortex rings contain highly three dimensional flow components, their main flow features can be analyzed and captured in one plane.^14^,^39^ Finally, the number of performed experiments was low, however, results were highly consistent and differences between the investigated groups were clearly visible, which is also supported by the statistical differences between the different support types for several parameters (see Table 2).

In conclusion, flow patterns in the LVOT during LVAD support were visualized and quantified in an *ex vivo* model using Echo-PIV for the first time. Altered ventricular flow patterns during mechanical circulatory support were observed with a diminished washout of the LVOT associated with higher degrees of LVAD support, potentially leading to stagnation areas that may contribute to adverse thromboembolic events.
